# Development and Implementation of a Web-Based Learning Environment for an Inpatient Internal Medicine Team: Questionnaire Study

**DOI:** 10.2196/18102

**Published:** 2020-12-29

**Authors:** Thaddeus Y Carson, Christos Hatzigeorgiou, Tasha R Wyatt, Sarah Egan, Sary O Beidas

**Affiliations:** 1 Augusta University Augusta, GA United States; 2 Sarasota Memorial Health Care System Sarasota, FL United States

**Keywords:** inpatient internal medicine, academic hospitalist, medical education, blended learning environment, social media, online education, internal medicine ward, internal medicine education

## Abstract

**Background:**

The notion of anytime, anyplace communication is characteristic of the current generation of learners. Such communications have facilitated the growth and integration of a blended or hybrid learning platform in multiple educational settings. However, there are limited reports on the use of an anytime, anyplace communication platform in clinical inpatient medical education.

**Objective:**

The setting of a high-demand inpatient clinical rotation is ideal for the use of collaborative software, and this integration is expected to positively influence medical education. The purpose of this study is to evaluate medical students’ and residents’ educational experiences with incorporating a simple, web-based content management and file sharing platform into an internal medicine inpatient rotation.

**Methods:**

During an inpatient internal medicine rotation, faculty and learners jointly used collaborative software for educational purposes, and a postrotation survey tool was used to measure the educational influence of the software.

**Results:**

Based on the results of the postrotation survey, the integration of a collaborative software application during clinical rotations improved the learning experience. Learning climate, the communication of rotation goals, and self-directed learning all scored favorably, but feedback from the survey participants was mixed. The learners enthusiastically accepted the practical use of this tool for both communication and information sharing.

**Conclusions:**

This generation of learners is accustomed to frequent electronic communication. Based on our survey, these learners appear to be highly receptive to this web-based intervention design for improving clinical education during active patient care. Adding effective blended learning features to a traditional clinical setting is achievable.

## Introduction

Bedside teaching is a fundamental component of medical training in the United States [1]. At the bedside, attending physicians are able to model clinical skills related to effective patient communication, clinical reasoning, and professional behavior. Although recent studies have indicated that bedside teaching may be on the decline [2,3] due to limitations with physicians’ time [4], this form of teaching remains an important part of medical training. Furthermore, although studies have investigated care providers’ perspectives on bedside teaching [5], learners’ perspectives are not often taken into account [6]. It is known that learners desire great flexibility in learning the critical skills, attitudes, and behaviors expected of physicians [1], in part because the notion of anytime, anyplace web-based communication is an expectation of the current generation of learners. Web-based communication allows team members to communicate in ways that are not always possible due to busy clinical schedules. Although the incorporation of such web-based spaces into in-service rounds appears to be a feasible solution for augmenting the inpatient teaching and learning environment, the integration of web-based spaces into clinical rotations is not well reported in the literature [1].

The purpose of this study is to evaluate medical students’ and residents’ educational experiences with incorporating a simple, web-based content management and file sharing platform into an internal medicine inpatient rotation. We hypothesized that by incorporating a web-based content management and file sharing platform into the rotation, learners would be better oriented to the expectations in the rotation, communication among team members would be streamlined, and learners would take more ownership over their educational processes.

## Methods

Augusta University [7] uses multiple learning systems to manage content and resources for different groups of learners. The use of several learning management systems ensures that no system is able to combine learners into a single clinical team or group. In an effort to overcome this challenge and improve the learning environment of the inpatient internal medicine ward teams, the authors integrated the institution’s software, Box, into internal medicine inpatient rotations.

Box is a cloud-based content management and file sharing service for businesses, and it is used by many medical schools for file sharing [8]. Several other platforms were initially considered, including the institution’s Learning Management System, Desire to Learn, a Share-Point site, and a departmental hard drive. However, the limitations of Desire to Learn were well recognized in the institution, largely because it was treated as a repository of content that was highly cumbersome for learners and not user-friendly. Additionally, the Share-Point site and institutional hard drive could not address issues related to the timely enrolling and unenrolling of learners, as these processes were managed at a departmental level.

The best option appeared to be Box, because it provided a means to share updated content and resources in an asynchronous learning environment within a group of invited users. It also afforded the opportunity for educators to manage various learner groups and provided institutional and user protection for protected health information. At the beginning of each rotation, all team members, including attending physicians, residents, interns, pharmacists, and third-year and fourth-year medical students, were enrolled into the Box folder. This study was designed to evaluate the strengths and weaknesses of this platform based on the perspectives of learners who participated in the rotation. These data were obtained using a qualitative survey questionnaire.

**Figure 1:**
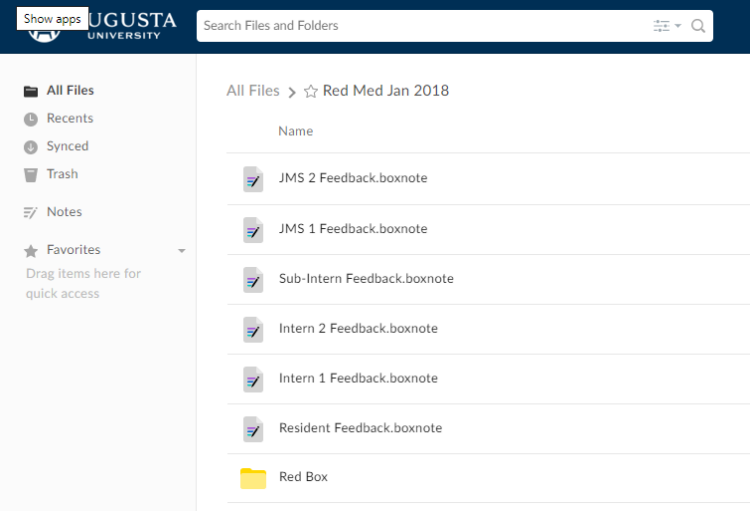
Screenshot of a Box folder from the first day of rotation. This screenshot displays the content that can be found in the "Red Med Jan 2018" section. Each Box note can only be accessed by the attending physician and the individual learner for private feedback and discussion.

**Figure 2:**
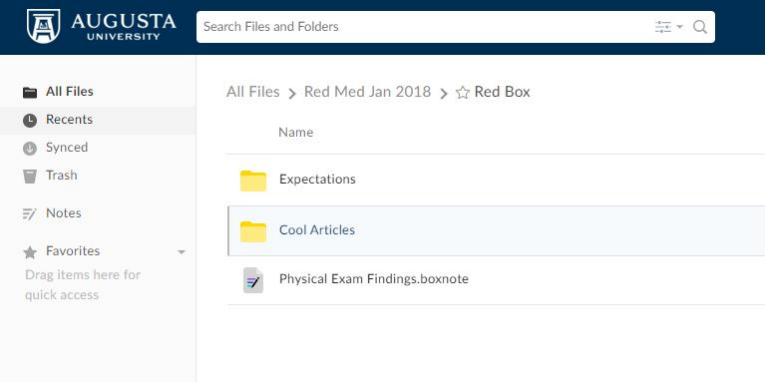
Screenshot of a Box folder from the first day of rotation. This screenshot displays the content of the "Red Box" section and is accessible to all learners.

On the first day of the rotation, students and residents were oriented to the features of Box, including the creation of folders to share resources with others on the ward team. Learners were also oriented to the attending physicians’ specific expectations for the rotation. Learners were instructed on where to store their presentations and where to find handouts for various disease and treatment processes that the attending physicians developed. Figure 1 and Figure 2 show the Box folders from the first day of the rotation. Other folders included a place for sharing patient information, such as interesting physical exam findings. This feature was useful for capturing important information when the time for bedside teaching was attenuated.

Learners were also oriented to the “Box notes” feature, which was used to communicate personalized feedback. The physicians created individual notes for each student and provided students with formative feedback on their presentations and patient write-ups. Learners were also oriented to the ability to leave “comments” in Box. This feature was used to create ongoing discussions on a particular topic, such as learners’ patient observations and evidence provided in uploaded journal articles or discussions on why medications were changed, in an asynchronous environment. These discussions could then be accessed by the team on their own time.

To evaluate learners’ perceptions on the efficacy and utility of Box, a short 15-item survey was created. This survey asked about learners’ experiences with using the web-based space provided by Box for collaboration. Items were written to assess learner attitudes toward Box, the use of Box as a learning platform, and the influence that Box has on the learning climate. The items also asked about whether Box assisted learners with understanding rotation expectations, promoted learner feedback, and encouraged self-directed learning. At the end of the survey, an open-response space was provided for additional comments.

The survey was electronically distributed to 67 students and residents via Qualtrics [9] at the end of 13 clinical rotations. Of the 67 surveys sent out, 44 were completed, providing a 66% response rate for survey completion. Data were then analyzed using descriptive statistics. A conventional content analysis was performed to categorize open-text responses into related comment groups [10]. Conventional content analyses are useful for when researchers are only interested in gaining an overall view of the present themes in textual data instead of applying a conceptual or theoretical framework to the study.

## Results

**Figure 3:**
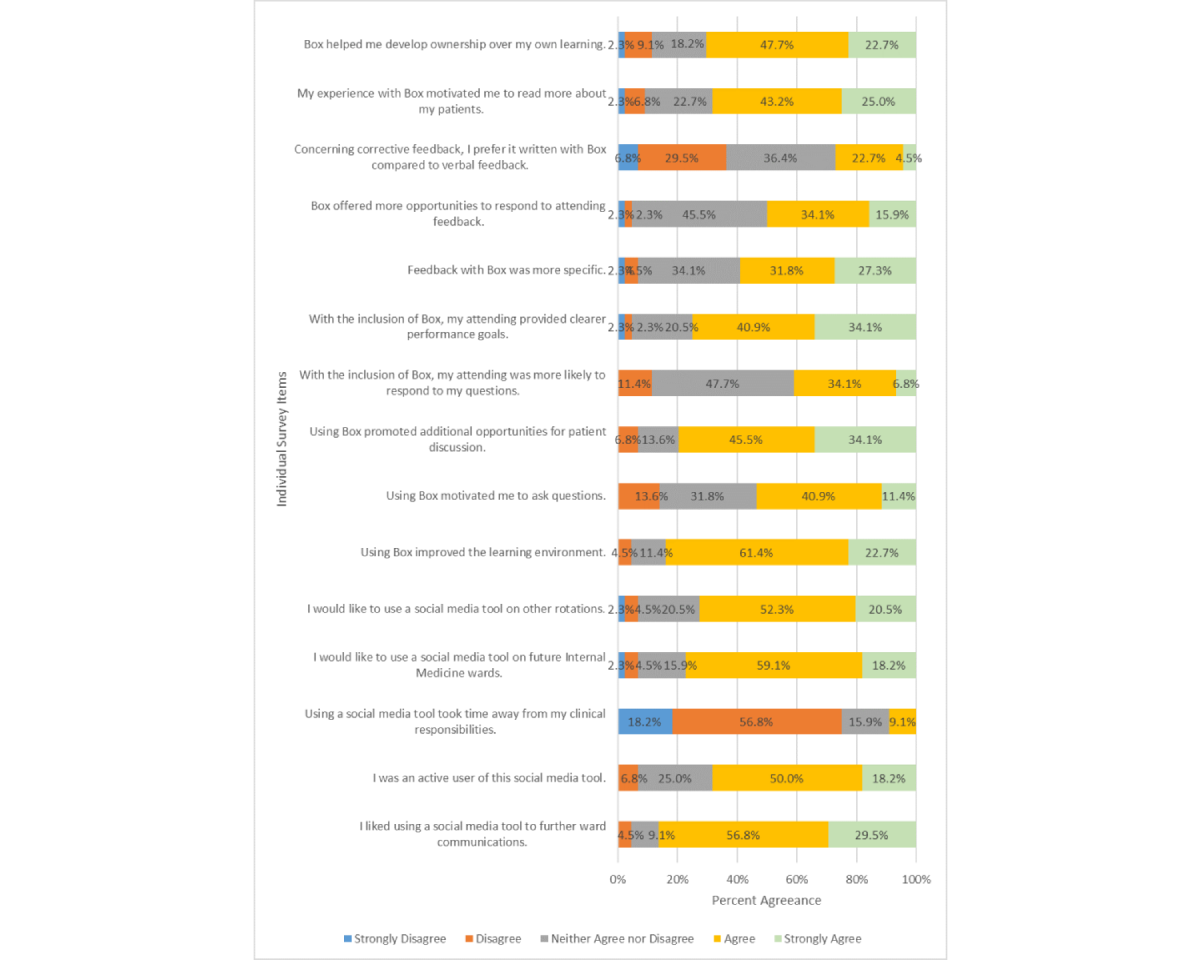
Postexperience survey tool responses.

Of the 44 participants who completed the study, 19 (43%) indicated that they were medical students, 12 (27%) indicated that they were residents, and 13 (30%) did not provide their role. There was no statistical difference between the number of medical students and residents across the different clinical groups across the following 4 subscales: the use of the tool (*P*=.31), the learning environment (*P*=.91), feedback (*P*=.91), and self-directed learning (*P*=.70). Overall, learners rated the use of Box within the internal medicine rotation with favorable responses for the following domains: attitudes toward the tool, the improvement of the learning environment, understanding expectations, receiving feedback, and self-directed learning (Figure 3).

The end-of-survey comments indicated that students and residents found the cloud-based collaborative tool useful for creating a web-based community and sharing resources with the team. The following is a comment from a student that represents the perspectives that were shared in the open-text response area: “This is an effective and more efficient means for feedback and focused education through group discussion and readings. All Internal Medicine rotations should use this tool.” However, some comments indicated that participants had the desire for even more feedback on presentations, such as the following: “Would have appreciated [more] feedback on presentations posted to the Box; good resource for team unity.” Surprisingly, there was only 1 negative comment made by a resident, who suggested that the use of Box could become an expectation and would add to residents’ growing areas of responsibility. The resident stated, “I would like to have to Box available as a resource, but there is a risk that there will be an unwritten expectation to keep up to date with the material placed in the Box. I believe this social site would best function in the background of the team and not in the center of the team.”

## Discussion

Based on the postrotation survey, the integration of a collaborative software application during a clinical rotation improved the learning experience. Learning climate (n=37, 84% agreeance), the communication of goals (n=33, 75% agreeance), and self-directed learning (n=31, 71% agreeance) all scored favorably, but feedback from the survey participants was mixed. The learners accepted the practical use (n=38, 86% agreeance) and feasibility (n=30, 68% agreeance) of this communication tool, and the supplemental process of information sharing (n=35, 80% agreeance). The mixed feelings in participant feedback could be explained by learners not understanding the intended meaning behind providing feedback in this context. Learners may have also believed that feedback needs to be delivered face-to-face [11].

To our knowledge, this is the first study to examine the value of using a software application to supplement and enhance the learning environment during clinical inpatient rotations. Published literature that describes the use of collaborative applications in nonclinical education, such as wikis and other web-based applications in medical education, are abundant [12]. However, using these tools in clinical rotations to complement the face-to-face learning and administrative functions of a clinical team has not been studied before [1,13,14].

**Figure 4:**
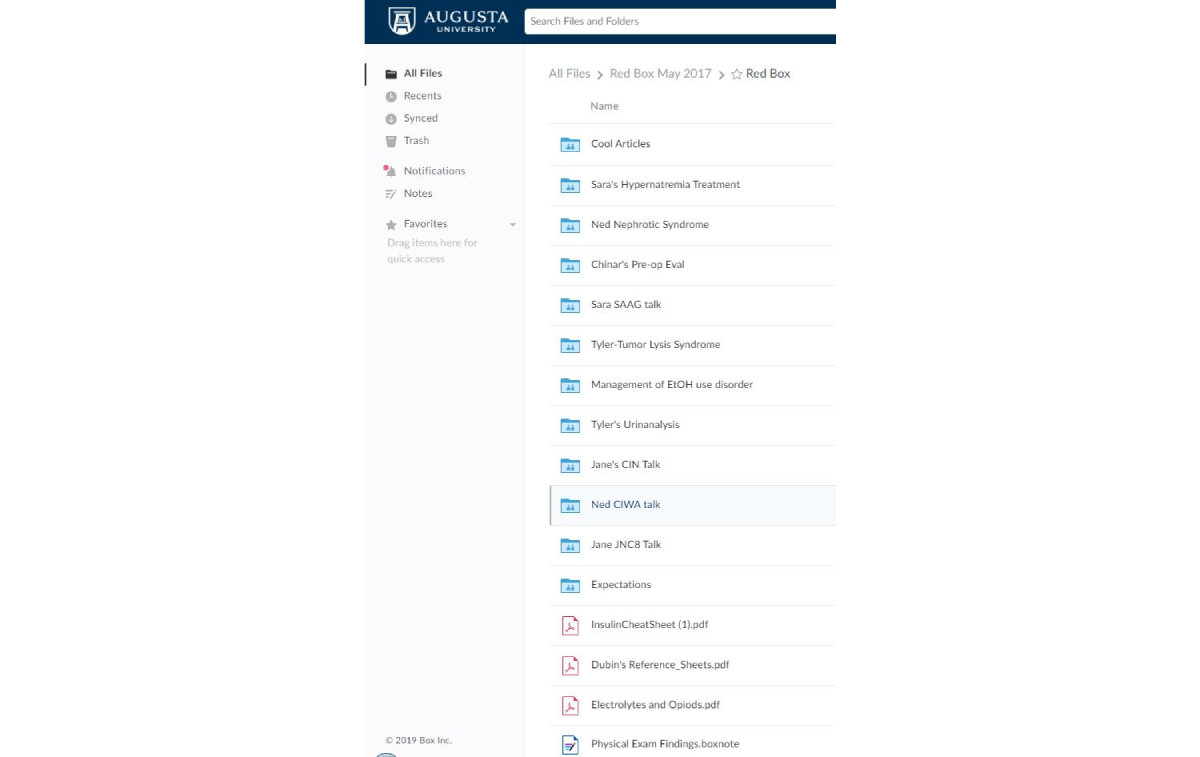
Screenshot that shows an example of what the Box folder looks like at the end of a rotation.

In this study, we emphasize the importance of using real patients that learners are connected and currently engaged with while providing medical care. This approach differs from other learning platforms that use simulated or virtual patients [1]. We were also able to capture salient learning events and documentation on learning activities within the team. Due to the constraints of resident availability (eg, days off, patient admissions, or other obligations), learners were able to access the application at their own convenience and participate in a myriad of ways, including posting discussions, engaging in reading related to patients, receiving feedback, and reflecting on their clinical performance. Figure 4 shows an example of what the Box folder looks like at the end of a rotation. We believe that by using Box, we were able to simplify the rotation environment and address what researchers refer to as “opening the black box” of the dissonance between what learners need and what educators deliver [15].

Overall, we believe that this generation of learners expect electronic communication. Based on our survey, these learners seemed to be highly receptive to this web-based intervention as a means of improving clinical education during active patient care. Additionally, we discovered that by referencing the Box entries, we were able to enrich the learners’ final evaluations with objective data for their formal evaluation and provide substantive comments for learners’ next steps. Future studies should focus on assessment strategies for capturing learner gains in terms of knowledge, skills, attitudes and behaviors.

We acknowledge that this study has several limitations. First, the survey was developed by the study team and was not assessed for validity evidence [16]. Future studies should consider collecting data to examine the survey for content, criterion, and construct validity. Second, response bias [17] from participants may have influenced participant responses. However, this would be challenging to assess unless the study was carried out with a different team that did not attend to respondents.
